# On the Use of Chloroform, and Ether in Dental Operations

**Published:** 1865-08

**Authors:** Henry S. Chase

**Affiliations:** Iowa City, Iowa


### ON THE USE OF CHLOROFORM AND ETHER IN DENTAL OPERATIONS.

BY HENRY S. CHASE, M. D., D. D. S., IOWA CITY, IOWA.

[Read before the Iowa State Dental Society.]

I believe that Chloroform and Ether are getting more and more into disuse every year, among dentists. Fifteen years ago, I used one or the other of them nearly every day. Patients *demanded* an anæsthetic of some kind. I have continued their use until the present time without any fatal results, and so far as I know, without serious results falling short of death or an impaired constitution. The most unpleasant effects from my administration of these, was from exhibiting Sulphuric Ether to a middle-aged maiden lady, who was indisposed (not dangerously) for two weeks. Other unpleasant effects were vomiting during the operation, if taken soon after eating.

We all know that Sulphuric Ether was the first anæsthetic introduced to the world. It soon became " all the rage" with those who wished surgical operations performed, and continued in almost universal use until Chloroform became a rival. During these years of the extensive and exclusive use of Ether, no serious accident resulted from its employment. But Chloroform very soon eclipsed its elder sister, and the latter was soon laid aside to make room for its highly favored rival. This continued until a sudden death, occurring now and then, startled the public like a thunderbolt. Some timid ones returned to Ether, but the great majority of surgeons and patients continued to use Chloroform. Its far more agreeable effects account for this, and its fascinations are sufficient to overcome the fear of death : the chance of this being estimated at only one in two hundred thousand cases.

Now I do not *advocate* the use of either Sulphuric Ether or Chloroform in the extraction of teeth. But when a proper subject demands it, I do not refuse. It is true that I prefer not to use either of them—*not* from a fear of fatal results, but from the *inconvenience* arising from its exhibition in this operation.

My choice in an anæsthetic for the extraction of teeth, is in favor of Chloroform ; but for a prolonged operation, say of several hours, it would be for Sulphuric Ether. The latter seems to be *entirely* safe. I have kept a patient unconscious most of the time for six hours together under its influence : it seems to leave no after depressing effects. On the contrary, Chloroform, when pushed for an hour or longer, is apt to leave the system very mnch reduced in its vital energy, and the reaction to a normal condition is postponed for many hours, sometimes to even many days.

But Ether is apt to *excite* the nervous too much in the commencement, and where *time* is an object, is objectionable on account of the lengthened period which it often requires to make the patient succumb to its influence. *Perfect quiet* is particularly desirable in the extraction of teeth; and this we cannot be at all sure of under the effects of Ether. Chloroform manifests its sedative powers immediately, and is con tinued for any length of time we desire.

Anæsthetics are contra-indicated in diseases of the heart, lungs and brain; and of course no operator would be foolhardy enough to exhibit it without previous inquiries or examinations in this respect.

I think I have the best results when using equal parts by measure of Chloroform and Sulphuric Ether combined.

Abundance of fresh air inhaled with these is all important. I have discarded all sorts of *apparatus*, and use a clean piece of sponge, rolled up in a napkin, with *both* ends open. I apply it to the open mouth and nose during inspiration, and remove it a little one side during expiration, so that it may not be contaminated by the life-spent air from the lungs.

No person should attempt this operation without a third person being present—not merely as an assistant, but as a witness. I think dentists have had sufficient warning as to this matter, without reasons being *now* adduced.

If many teeth are to be extracted at one sitting, it is very desirable to have an experienced operator administer the anæsthetic, and another equally experienced to extract the teeth. But if there are only two or three teeth to be removed, almost any person of calm temperament can sufficiently assist the dentist.

Let every necessary preparation be made *before* commencing.

Fresh water, Brandy or Alcohol, Ammonia, a fan, napkins and towels, are all necessary.

Place your extracting instruments within reach, properly *selected.*

Styptics, for arresting hemorrage, should not be forgotten; for the less blood your patient has to evacuate from the mouth, the better.

Commence very gradually with the vapor; allow the lungs to get accustomed to it before attempting to crowd it.

Note the condition of the pulse before the patient is seated in the operating chair.

And here let me remark, that an upright position is all important in *this* operation. I would prefer that a patient should lean *forward,* rather than back; otherwise, strangulation might occur from the presence of blood.

If the pulse continues at its normal standard, or increases in volume and frequency under the effects of the vapor, I consider it a favorable indication. But if it falls below the natural condition, I should immediately cease its exhibition, and apply restoratives. Wait a few minutes, and try again. But if the same unfavorable symptoms recur, I should refuse the anæsthetic altogether. There would not, however, be any objection to a trial of Sulphuric Ether alone, under these circumstances.

If your patient will consent to extraction in a state short of unconsciousness, by all means take advantage of it. The greater number will agree to it, and will tell you when they "have enough" of the vapor.

You must let your patient recover often enough to clear the mouth of blood. As soon as the operation is through, unless consciousness has returned, sprinkle the face and back of the neck with water, use the fan, and apply Ammonia to the nose.

Among the preliminaries, I ought to have stated that the dress should be perfectly loose over the lungs and stomach, so that the lungs and diaphragm can have full play. And to facilitate this, it is better that the stomach be nearly empty. A full stomach is to be avoided, certainly, for otherwise vomiting is almost certain to happen.

When fully under the influence of the vapor, breathing *almost* ceases; and at this point, of course, if not before, the extraction should commence.

I have never had a case where respiration *entirely* ceased. In such a condition, *artificial* respiration and friction are recommended. All those movements, in fact, that are used for the resuscitation of drowned persons. Energetic efforts should not be relaxed for hours after apparent death. Dr. J. H. McQuillen has demonstrated that the action of the heart in a rabbit continued two hours and a half after artificial respiration had ceased. Doubtless it continues a long time after the contraction of the arteries can be felt in the pulse. With these facts before us, no one would be justified in relinquishing his efforts for resuscitation under an hour or two of persevering effort.

In nearly all fatal cases from the administration of Chloroform, it has been noticed that death ensued *before* the patient was brought under its desired influence. Paralysis of the heart seemed to occur; a sudden failure of the pulse, and the patient apparently dead. On some occasions, the patient had previously taken the vapor with kindly results—sometimes only the day before.

How such untoward results are to be avoided, I cannot say. In my opinion, it is no valid reason why Chloroform should never be exhibited, any more than it would be to resolve to never ride again in a railroad car, because fatal accidents occasionally take place on them.

After extraction, the patient should be allowed to lie down and sleep, reposing on the side, with napkins, for wiping the mouth, at hand.

Anæsthesia may be prolonged with safety with a much less quantity of the vapor, by giving the patient a half hour beforehand a full dose of morphia. However, in the extraction of teeth, you must judge whether it is a case in which you would like its *continued* effect, for in the exhibition of the narcotic in connection with that of the vapor there will be no intervals of consciousness, until the whole effect has passed away.

These vapors should never be exhibited immediately after the patient has taken alcoholic stimulants. I have been occasionally annoyed in this way, by men taking a glass of brandy to give them courage to inhale the anæsthetic. The result is, much nervous excitement and difficulty in producing anæsthesia. The stimulant has the effect to destroy the action of the vapor. Under such circumstances, I have witnessed symptoms of insanity for a short time, and resistance to the operation is manifested by the patient.

I find women and children more kindly affected by anæsthesia than men. It is peculiarly favorable in its effects on children. I do not remember of a death ever having taken place when administered to a child.

There is nothing *new* in what I have said on this subject; but these remarks are made for the encouragement of those whose *fears* have prevented them from having actual experience in the use of anæsthetics. Dentists have many more occasions for their use than practitioners of medicine, and they ought, for their own reputation, and that of the profession in general, to become well acquainted with their use.

